# Sudden Death in Infectious Diseases

**Published:** 1908-05-23

**Authors:** 


### Diseases of Children.

### SUDDEN DEATH IN INFECTIOUS DISEASES.

It is by no means rare for sudden or unexpected death to occur during the course of an infective disease, even though apparently of a mild type and running a normal course. The end may come with appalling suddenness, the child sitting up in bed and falling back dead, or may be preceded by a period of profound collapse with dilated pupils and rapid feeble cardiac action, the breathing remaining little or not at all affected. It is customary to ascribe the death to heart failure, a term beloved by the public as well as the profession, and often erroneously used. Moreover, there is no doubt that many would ascribe the result to myocardial affection. But on post-mortem examination it will be found that in those cases in which death has occurred in the first week of an attack of a croupous pneumonia, scarlatina, diphtheria, or such like disease, and has taken place after a period of collapse, there is neither macroscopical nor microscopical evidence of any change in the heart muscle. There is no dilatation of the right side of the heart, which, as a whole, is found in an empty and contracted state. There is no evidence whatever of heart failure in the true acceptation of the term. The stage of collapse is much more analogous to that of surgical shock or severe hæmorrhage, and is, in all probability, due to paralysis of the vasomotor centre in the bulb, leading to a rapid and profound fall in blood pressure and death from the so-called hæmorrhage into the splanchnic area. Thus, the characteristic features of this mode of death are the rapid fall in blood pressure, weak and empty pulse, pallor, and collapse. Treatment must be directed to raising the blood pressure. The injection of barium chloride is useless, for it raises pressure by its action on the muscular coats of the small arteries and the peripheral nerves. Digitalis may do good by its action on the vasomotor centre and peripheral vessels, so too hypodermic injections of atropine. Cold packs to the abdomen or the cold bath may do good by their stimulating effect on the vasomotor centre, and are of use in prophylaxis, but when once the condition has started there is little hope, and we must rely mainly on the recumbent posture with the foot of the bed well raised and the head low, stimulants such as caffein and strychnia, and subcutaneous injections of normal saline and adrenalin.

In the other variety of sudden death there is distinct evidence of myocardial disease, most marked in diphtheria, severe scarlatina, and acute rheumatism. In high fever the heart muscle may be soft and friable as early as the fourth day from cloudy swelling, and a general myocarditis may develop. The first indication is irregularity, and it is followed by all the symptoms and signs of cardiac dilatation. Post-mortem there is usually extensive fatty degeneration of the muscle and acute degeneration in certain cells of the central nervous system, in the anterior cornua and the motor nucleus of the vagus in cases of diphtheria. This type of cardiac affection is now well recognised and described as myocarditis. Everyone should be on the watch for it in infective diseases, especially diphtheria and acute rheumatism. The treatment is that of myocarditis, mainly, complete rest in the recumbent posture, cardiac tonics, and careful feeding, avoiding indigestible food and over-distension of the stomach. The prognosis is comparatively good in children, but very great care is essential and often most prolonged treatment, sometimes for several months. Increase in blood pressure, due to nephritis, acting on a dilated heart, has been known to cause sudden death in scarlatina.

Bulbar palsy also occurs in diphtheria and other infections, and occupies a position closely related to the vasomotor palsy above described. It is a cause of cardio-pulmonary paralysis, dyspnœa, and rapid death, due to the direct action of the toxin on the bulbar centres. It produces dyspnœic attacks which have been named by Guthrie "Bulbar Crises." Such attacks may occur every few minutes or hours, and end in death from exhaustion, syncope, asphyxia, or cardiac thrombosis. The duration of each attack is very variable. The early signs are irregular, sighing breathing, accumulation of mucus in the air passages, and rapid pulse. In the attacks these signs are much exaggerated, and there is profound listlessness and apathy. They may come on without apparent cause or may be induced by exertion or excitement. In a bad attack there are alarming dyspnœa, distended alæ nasi, open mouth, dilated pupils, bluish pallor, and sweating skin. The breathing is irregular, gasping or sighing, and there is a general accumulation of mucus. The pulse is much increased in frequency, weak and small. The temperature is raised. Occasionally an attack ends in severe vomiting. In addition there may be sudden and complete paralysis of deglutition, aphonia, and paralysis of the diaphragm.

Vagus paralysis is a further cause of sudden death. Strictly speaking, this is a part of bulbar palsy, for the paralysis of the vagus due to degenerative changes in the nerve, apart from nuclear palsy, is more likely to end fatally through the secondary cardiac degeneration to which it gives rise. Cardiac thrombosis may prove suddenly fatal. Here, too, it is more likely to occur as a complication of cardiac dilatation than as the primary cause of death.

Thus we may look upon sudden or unexpected death in the course of infectious disease as the result of a pure paralysis of the vasomotor centre in the medulla, analogous to the failure of this centre to respond to stimuli in the case of surgical shock; as occasionally the result of bulbar palsy; and as due to the myocarditis which may be set up even in the early stages of the disease by the action of the toxins on the heart muscle, and, later on, produced in a similar manner or secondary to the degenerative changes in the nerve cells in the spinal cord or bulb and in the nerves. A due understanding of these differences in the mode of production of sudden death will lead to a more accurate use of the term heart failure in medical literature.

